# Instrumental Divergence and the Value of Control

**DOI:** 10.1038/srep36295

**Published:** 2016-11-04

**Authors:** Prachi Mistry, Mimi Liljeholm

**Affiliations:** 1 Department of Cognitive Sciences, University of California, Irvine, USA

## Abstract

A critical aspect of flexible choice is that alternative actions yield distinct consequences: Only when available action alternatives produce distinct outcome states does discrimination and selection between actions allow an agent to flexibly obtain the currently most desired outcome. Here, we use *instrumental divergence* – the degree to which alternative actions differ with respect to their outcome probability distributions – as an index of flexible instrumental control, and assess the influence of this novel decision variable on choice preference. In Experiment 1, when other decision variables, such as expected value and outcome entropy, were held constant, we found a significant preference for high instrumental divergence. In Experiment 2, we used an “auto- vs. self-play” manipulation to eliminate outcome diversity as a source of behavioral preferences, and to contrast flexible instrumental control with the complete absence of voluntary choice. Our results suggest that flexible instrumental control over decision outcomes may have intrinsic value.

The ability to exert flexible control over one’s environment is a central feature of adaptive decision-making. One critical aspect of flexible choice is that alternative actions yield distinct consequences: If all available action alternatives have identical, or similar, outcome distributions, such that selecting one action over another does not significantly alter the probability of any given outcome state, an agent’s ability to exert flexible control over its environment is considerably impaired. Conversely, when available action alternatives produce distinct outcome states, discrimination and selection between actions allows the agent to flexibly obtain the currently most desired outcome. Notably, since subjective outcome utilities often change from one moment to the next (e.g., due to sensory satiety), flexible instrumental control is essential for reward maximization and, as such, may have intrinsic value. Here, we use *instrumental divergence* – the degree to which alternative actions differ with respect to their outcome probability distributions – as an index of flexible control, and assess the influence of this novel decision variable on behavioral choice preference.

Formal theories of goal-directed control postulate that the agent generates a “cognitive map” of stochastic relationships between actions and states such that, for each action in a given state, a probability distribution is specified over possible outcome states. These transition probabilities are then combined with current estimates of outcome utilities in order to generate action values – the basis of goal-directed choice^1^,^2^. The separate estimation and “on-the-fly” combination of outcome probabilities and outcome utilities offers adaptive advantage over more automatic action selection, which uses cached values based on reinforcement history^1^. There are, however, situations in which goal-directed computations do not yield greater flexibility.

As an illustration, consider the scenario in Fig. 1a, which shows two available actions, A1 and A2, with bars representing the transition probabilities of each action into three potential outcome states, O1, O2 and O3. Here, the goal-directed approach prescribes that the agent retrieves each transition probability, estimates the current utility of each outcome, computes the product of each utility and associated probability, sums across the resulting value distribution for each action and, finally, compares the two action values^1^. Of course, given equivalent costs, actions that have identical outcome distributions, as in Fig. 1a, will inevitably have the same value, eliminating the need for resource-intensive goal-directed computations. However, critically, this lack of instrumental divergence also eliminates the power of choice: selecting A1 over A2, or vice versa, does not alter the probability of any given outcome state.

Now consider the scenario in Fig. 1b, in which the probability distribution of A2 has been reversed across the three outcomes, yielding high instrumental divergence. Note that, if the utilities of O1 and O3 are the same, the two actions still have the same *expected value*. Likewise, *outcome entropy* – the uncertainty about which outcome will be obtained given performance of a particular action – is the same for both actions. In spite of this equivalence, the two actions in Fig. 1b clearly differ. To appreciate the significance of this difference, imagine that O1 and O3 represent food and water respectively, and that you just had a large meal without a drop to drink. Chances are that your desire for O3 is greater than that for O1 at that particular moment. However, a few hours later, you may be hungry again and, having had all the water you want, now have a preference for O1. Unlike the scenario illustrated in Fig. 1a, the high instrumental divergence afforded by action-outcome contingencies in Fig. 1b allows you to produce the currently desired outcome as preferences change, by switching between actions. Thus, instrumental divergence can serve as a measure of agency – the greater the divergence between available actions, the greater the degree of flexible instrumental control. Here, to assess a behavioral preference for flexible control, we use a novel paradigm in which participants choose between environments with either high or low instrumental divergence.

## Experiment 1

### Method

#### Participants

Twenty-four undergraduates at the University of California, Irvine (19 females; mean age = 20.42 ± 1.77) participated in the study for course credit. All participants gave informed consent and the Institutional Review Board of the University of California, Irvine, approved the study. All aspects of the study conformed to the guidelines of the 2013 WMA Declaration of Helsinki.

#### Task and Procedure

The task is illustrated in Fig. 2. At the start of the experiment, participants were instructed that they would assume the role of a gambler in a casino, playing a set of four slot machines (i.e., actions, respectively labeled A1-A4) that would yield three different colored tokens (blue, green and red), each worth a particular amount of money, with different probabilities. They were further told that, in each of several blocks, they would be required to first select a “room” in which only two slot machines were available, and that they would be restricted to playing on those two machines on subsequent trials within that block. Recall that instrumental divergence is a measure of the *difference* between actions with respect to their outcome probability distributions. Consequently, a preference for high instrumental divergence can only be assessed if each option contains at least two action alternatives. Here, the instrumental divergence of available slot machines differed across room options. The measure of interest, thus, was the decision at the beginning of each block (top of Fig. 2), between a high- versus low-divergence room. If flexible control, defined as high instrumental divergence, has intrinsic value, participants should prefer the high-divergence room, other things (e.g., expected monetary values and outcome entropy) being equal. To ensure that each room choice was consequential, participants were restricted to gambling on the slot machines available in the selected room for the duration of that block.

We were primarily interested in assessing a preference for high instrumental divergence when other decision variables were held constant. Thus, in the majority of blocks, identical monetary pay-offs were associated with high- and low-divergence rooms. However, we also included subsets of blocks in which monetary pay-offs differed across rooms, in either the same or opposite direction of instrumental divergence: These additional blocks served to confirm that participants in our task were sensitive to differences in expected monetary value, allowing us to interpret their performance in terms of conventional theories of reinforcement learning and economic choice. Note that, when instrumental divergence and expected monetary pay-offs differ in the same direction across rooms, both variables predict selection of the same room (i.e., that with greater divergence *and* a greater monetary pay-off), in an additive fashion. In contrast, when these two variables differ in opposite directions, so that the room with the greater monetary pay-off is that with *lower* instrumental divergence, they compete for control of behavior (i.e., the greater monetary pay-off is pitted *against* the value of high instrumental divergence). Consequently, we predicted that participants would be more likely to select the room with a greater expected monetary pay-off when that room was also associated with high instrumental divergence than when it was associated with low instrumental divergence.

**Figure 1:**
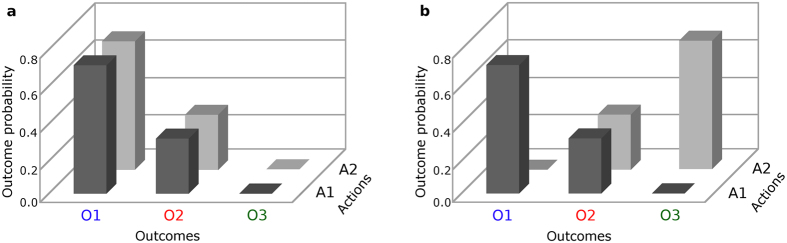
Probability distributions over three potential outcomes (O1, O2 & O3) for two available actions (A1 & A2) across which instrumental divergence – the difference between outcome probability distributions – is zero (**a**) or high (**b**).

Recall that it is *because* the subjective utility of a given outcome may change from one moment to the next (e.g., you may crave chocolate and then sate yourself on it, the value of a stock might increase one day and plummet the next) that flexible instrumental control is essential for reward maximization. Returning to the scenario illustrated in Fig. 1, if the utilities of O1 and O3 were identical and fixed, the high instrumental divergence afforded by the probability distributions in Fig. 1b would be of little consequence. On the other hand, if the utilities of O1 and O3 fluctuated, so that O1 was sometimes worth more and other times less than O3, the high instrumental divergence in Fig. 1b would allow an agent to maximize utility by switching between A1 and A2 according to current preferences. Here, to motivate the use of instrumental divergence as a decision variable, we simulate dynamic fluctuations in outcome utilities by changing the monetary values assigned to the different tokens at various points throughout the experiment.

#### Decision variables

**Figure 2:**
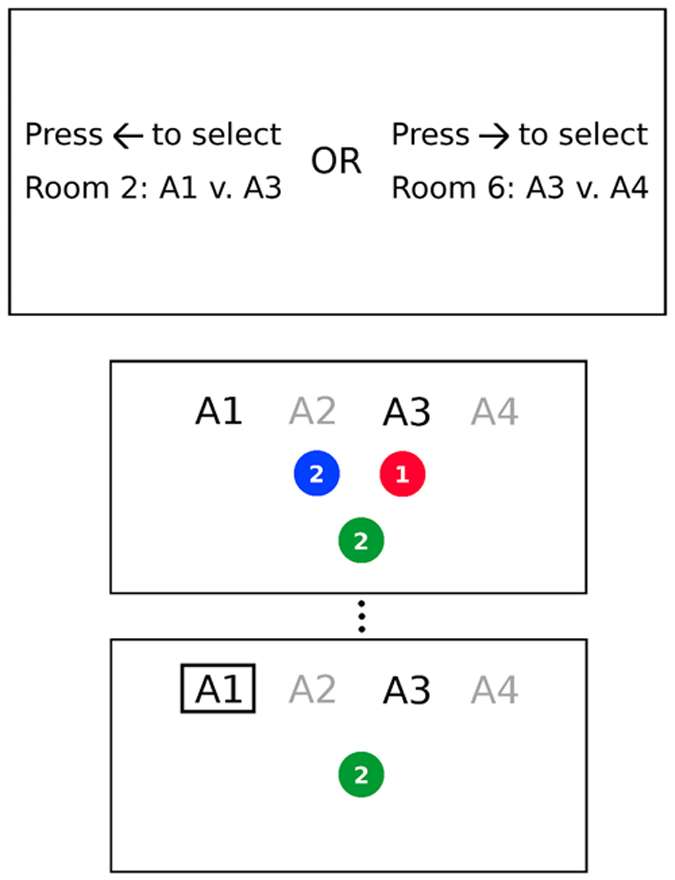
Task illustration showing the choice screen at the beginning of a block (top), and the choice screen (middle) and feedback screen (bottom) from a trial within a block.

Our measure of interest was the decision made at the beginning of each block, between two gambling rooms (i.e., action pairs; see top of Fig. 2) that differed in terms of instrumental divergence and, sometimes, expected value. We formalize the instrumental divergence of a gambling room as the Jensen-Shannon (JS) divergence^3^ of the token probability distributions for the two actions available in that room. Let *P*_*1*_ and *P*_*2*_ be the respective token probability distributions for the two actions available in a gambling room (e.g., A1 and A2), let *O* be the set of possible token outcomes (i.e., red, green and blue), and *P*(*o*) the probability of a particular (e.g., red) token outcome, *o*. The instrumental divergence of a gambling room is defined as:


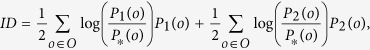


where


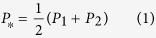


Thus, instrumental divergence is the mean logarithmic, symmetrized, difference between outcome probabilities for alternative actions. Note that, while we are comparing only two available actions, this divergence measure can be generalized to any finite number of probability distributions^3^, allowing for a comparison of many more action alternatives. Note also that instrumental divergence is defined here with respect to the sensory-specific (i.e., colors) rather than motivational (i.e., monetary) features of token outcomes, allowing for a clear dissociation of divergence and expected value.

We defined the *expected value* of a room as the sum over the products of outcome probabilities and outcome utilities given a particular action, summed over the two actions available in the room:


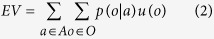


where *A* is the set of actions available in a room (e.g., A1 and A2), *O* is, again, the set of possible token outcomes, *p*(*o|a*) is the probability of a particular token outcome *o* conditional on a particular action *a*, and *u*(*o*) is the utility (i.e., monetary value) of outcome *o*.

Finally, an important decision variable frequently shown to influence instrumental choice is the variability, or entropy, of outcome states^4^,^5^,^6^, which is greatest when the probability distribution over outcomes is uniform. Given the actions available in a room, where *A*, *O* and *p*(*o|a*) are defined as above, and *p*(*a*,*o*) is the joint probability of action *a* and outcome *o*, the outcome entropy of that room is defined as:


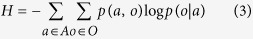


We did not manipulate the entropy of gambling rooms but define it here in order to specify that it was held constant across all room options throughout the task, at 0.88 bits (where a *bit* is the unit of information for logarithmic base 2, used in both equations 1 and 3). This allows us to eliminate outcome entropy as a source of any observed preference for one room over another.

#### Choice scenarios

In this section we outline the assignment of conditional probabilities and reward magnitudes to token outcomes, the pairing, given those assignments, of actions in high- versus low-divergence rooms and the combination of rooms into choice scenarios. The construction of choice scenarios is summarized in Table 1. We used two distinct probability distributions over the three possible token outcomes: [0.7, 0.3, 0.0 and 0.0, 0.3, 0.7]. The assignment of outcome distributions to actions was such that two of the actions shared one distribution, while the other two actions shared the other distribution. These assignments were counterbalanced across subjects, such that, for half of the subjects, A1 & A2 shared one distribution and A3 & A4 shared a different distribution (as in Table 1). For the remaining subjects, A1 & A3 shared one distribution and A2 & A4 shared the other (thus, contrary to the scheme in Table 1, for these participants, zero-divergence rooms contained A1 & A3 or A2 & A4). In both groups, this yielded a low (zero) instrumental divergence for rooms in which the two available actions shared the same probability distribution (as in Fig. 1a), and a high (0.7 bits) instrumental divergence for rooms in which available actions had different outcome probability distributions (as in Fig. 1b). The four actions were combined into six pairs (i.e., rooms), which were in turn combined into 10 two-alternative choice scenarios (as that shown in top of Fig. 2). For 8 of these scenarios, divergence differed across the two rooms, and each of these 8 scenarios were repeated 2 to 5 times, depending on expected value constraints discussed below, in random order across 28 blocks. For completeness, we also included two choice scenarios in which divergence was either equally low or equally high for both rooms. Each such scenario was repeated 4 times and distributed randomly among the other 28 blocks, yielding a total of 36 blocks. Each block consisted of 6 trials in which participants chose between the two actions in the selected room, for a total of 216 trials.

In the majority of blocks, the reward magnitudes assigned to the blue, green and red token respectively ($2, $2 and $1) yielded identical expected values for all actions. However, we also used token-reward assignments that yielded differences in expected value across rooms. Thus, in two subsets of blocks, the relative token values were such that the expected value of the zero-divergence room was either greater ($2.30) or lesser ($1.60) than that of the high-divergence room ($1.95). Transitions between token-reward assignments occurred every 3–5 blocks (every 4^th^ block on average), were explicitly announced, and always occurred after the participant had already committed to a particular room in a given block. We refer to blocks in which expected value was constant across rooms as balanced (B). Blocks in which expected value differed across rooms in the opposite direction of divergence are referred to as “unbalanced opposite” (UBO) and those in which expected value differed across rooms in the same direction as divergence as “unbalanced same” (UBS). For filler blocks, in which the two rooms had the same divergence, high or low, expected value was always balanced across rooms. For critical blocks, in which divergence differed across the rooms, 12 were B, 8 were UBO and 8 were UBS, with the order of B, UBO and UBS blocks counterbalanced across participants. Note that all monetary rewards were fictive, and that participants were instructed at the beginning of the study that they would not receive any actual money upon completing the study.

#### Pre-training on action-token probabilities

Before starting the gambling task participants were given a practice session in order to learn the probabilities with which each action produced the different colored tokens. To avoid biasing participants towards any particular reward distribution, no values were printed on the tokens in the practice session. To ensure equal sampling, each action was presented individually on 10 consecutive trials, with tokens occurring exactly according to their programmed probabilities (i.e., if the action produced green tokens with a probability of 0.3, the green token would be delivered on exactly 3 of the 10 trials). Following 10 trials with a given action, participants rated the probability with which that action produced each colored token on a scale from 0 to 1.0 with 0.1 increments. If the rating of any outcome probability deviated from the programmed probability by more than 0.2 points, the same action was presented for another 10 trials, and this process repeated until all rated probabilities were within 0.2 points of programmed probabilities for that action. After receiving training on, and providing ratings for, each action, participants were required to rate the outcome probabilities for all four actions in sequence; if the rating of any probability deviated from the programmed probability by more than 0.2 points, the entire practice session was repeated.

### Results

#### Pre-training on action-token probabilities

Participants required on average 2.17 (*SD* = 1.17) sessions of practice on the action-token probabilities. Mean probability ratings, obtained right before and right after the gambling phase, are shown in the top two rows of Table 2. On average, rated probabilities were very close to programmed ones, both prior to gambling, and immediately following the gambling phase.

#### A preference for high instrumental divergence

**Figure 3:**
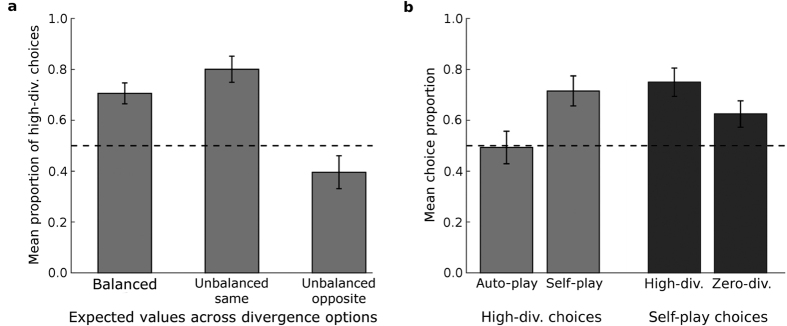
Mean choice proportions in Experiments 1 and 2. Dashed lines indicate chance performance. Error bars = SEM. (**a**) Mean proportions of high- over zero-divergence choices, for blocks in which expected values were identical across high- and low-divergence options (Balanced), blocks in which expected values differed across options in the same direction as divergence (Unbalanced same) and blocks in which expected values differed in the opposite direction of divergence (Unbalanced opposite), in Experiment 1. (**b**) Mean proportions of high- over zero-divergence choices (left) for blocks in which the high-divergence option was Auto-play versus blocks in which the high-divergence option was Self-play, and mean proportions of self- over auto-play choices (right) for blocks in which both options had high-divergence (High-div.) versus blocks in which both options had zero-divergence (Zero-div.), in Experiment 2.

The mean proportions of high-divergence over zero-divergence choices, for B, UBO and UBS blocks, are shown in Fig. 3a. Our primary hypothesis was that, when both expected value and outcome entropy were held constant across rooms (i.e., in Balanced blocks), participants would prefer the room with high instrumental divergence. Planned comparisons confirmed this prediction: For blocks in which instrumental divergence differed across rooms, while expected value and outcome entropy were held constant, the mean proportion of high-divergence over zero-divergence choices was significantly different from chance, *t*(*23*) = *5*.*00*, *p* < *0*.*001*, *d* = *1*.*02*. Critically, we confirmed that, consistent with programmed reward contingencies for balanced blocks, mean monetary earnings did not differ significantly across high- ($10.84 ± 0.56) and zero- ($10.72 ± 0.65) divergence rooms, *t*(*23*) = *0*.*53*, *p* = 0.60.

We further hypothesized that there would be a significant effect of expected monetary value, such that the proportion of high-divergence over zero-divergence choices would be greater when expected value differed across rooms in the same direction as divergence (Unbalanced same) than when expected value differed in the opposite direction (UBO). Since monetary rewards were fictive, these conditions provide important criterion checks, confirming that participants were sensitive to differences in expected monetary pay-offs. Consistent with a previously demonstrated correspondence between real and fictive monetary rewards, in both behavioral choice and neural correlates^7^,^8^,^9^, a planned comparison revealed that participants’ choices were indeed in accordance with expected monetary rewards: the proportion of high- over zero-divergence choices was significantly greater in UBS than in UBO blocks, *t*(*23*) = *4*.*88*, *p* < *0*.*001*, *d*_*z*_ = *1*.*00*. Finally, we predicted that, due to the competing effects of instrumental divergence and expected value, the deviation from chance performance would be greater in UBS than in UBO blocks, an asymmetry that is apparent in Fig. 3a. This prediction was confirmed: in spite of equal differences in absolute expected value, choice performance deviated significantly from chance when expected value differed in the same direction as instrumental divergence, *t*(*23*) = *5*.*86*, *p* < *0*.*001*, *d* = *1*.*20*, but not when expected value differed in the opposite direction of instrumental divergence, *t*(*23*) = *1*.*61*, *p* = *0*.*121*, *d* = *0*.*34*.

## Experiment 2

The results of Experiment 1 confirm that, when given a choice between environments that have either high or zero instrumental divergence, participants strongly prefer the high-divergence option. We interpret this preference as reflecting the intrinsic value of flexible instrumental control. Alternatively, however, participants’ choices may reflect a previously demonstrated tendency to maximize *outcome diversity* – the perceptual distinctiveness of potential outcomes^10^,^11^. Although highly related, in that greater instrumental divergence may yield greater outcome diversity, as was the case in Experiment 1, the flexible control afforded by divergence does not follow from outcome diversity.

In zero-divergences rooms in Experiment 1, illustrated in Fig. 1a, regardless of which action was selected, there was a high probability of obtaining O1, a low probability of obtaining O2 and a zero probability of obtaining O3 (where each numbered outcome indicates a distinctly colored token). In contrast, in high-divergence rooms, illustrated in Fig. 1b, participants were able to obtain *both* O1 and O3, as well as O2, by switching between actions across trials. Consequently, even when the expected values of high- and zero-divergence rooms were identical, as in the majority of blocks in Experiment 1, the perceptual diversity of obtainable outcomes was greater in high- than in zero-divergence rooms (i.e., three differently colored tokens were obtainable in high-divergence rooms, but only two in zero-divergence rooms). It is possible, therefore, that the preference for high instrumental divergence found in Experiment 1 reflects a previously demonstrated preference for greater perceptual diversity among obtainable outcomes^10^,^11^. Now, consider a scenario in which a computer algorithm chooses between the actions in a given room, selecting each action equally often by alternating across trials. In this case, the high-divergence room would still yield greater outcome diversity than the zero-divergence room; however, in the absence of voluntary choice, the high-divergence room no longer yields flexible instrumental control. Indeed, in the absence of free choice, neither the high- nor zero-divergence condition can be considered *instrumental*.

Our main hypothesis is that greater instrumental divergence is valuable because it yields greater levels of flexible instrumental control. When the instrumental component is removed, as when a computer selects between actions while participants passively observe, so is the potential for flexible control. Consequently, we do not predict any preference for high divergence rooms in the absence of free choice. However, a computer algorithm selecting both actions in a room equally often, by alternating across trials, would ensure that the diversity of obtained outcomes is still greater in high- than in zero-divergence rooms. Therefore, if choices in Experiment 1 were driven by a desire to maximize outcome diversity, rather than instrumental divergence, similar preferences should emerge whether the participant or an alternating computer algorithm choose between the actions in a room. In Experiment 2, we used an “auto-play” option, in which the computer selected between the two actions available in a room, to rule out outcome diversity as the source of a preference for flexible instrumental control.

### Method

#### Participants

Twenty-four undergraduates at the University of California, Irvine (19 females; mean age = 20.42 ± 2.62) participated in the study for course credit. All participants gave informed consent and the Institutional Review Board of the University of California, Irvine, approved the study. All aspects of the study conformed to the guidelines of the 2013 WMA Declaration of Helsinki.

#### Task and Procedure

In any given gambling room in Experiment 2, whether high or low in instrumental divergence, a computer algorithm selecting both actions equally often, by alternating across trials, would maximize outcome diversity in that room. Consequently, if the choice is between two rooms with equal levels of divergence, where one room is auto-play (the computer chooses between actions in the room) and the other is self-play (the participant chooses between actions in the room), outcome diversity maximization does not predict a preference for either room. Conversely, if the choice is between two rooms that *differ* in terms of divergence, outcome diversity maximization predicts a preference for the high-divergence room, since that is also the option with greater outcome diversity, whether it is associated with self-play or auto-play. In contrast, according to our hypothesis, that it is the flexible instrumental control afforded by high divergence that has intrinsic value, there should be a clear preference for the combination of high divergence and self-play. Thus, if choosing between two high-divergence rooms, one auto-play and the other self-play, there should be a preference for the self-play room. On the other hand, if choosing between two zero-divergence rooms, one auto-play and the other self-play, the preference for self-play should be much weaker, or absent, since zero-divergence rooms do not yield flexible control even under self-play conditions. Likewise, if choosing between a high- and a zero-divergence room, a preference for the high-divergence room should only emerge if that room is associated with self-play, since high-divergence rooms do not yield flexible instrumental control under auto-play conditions.

The task and procedure were identical to Experiment 1, with the following exceptions: First, in addition to potentially differing in terms of instrumental divergence and expected monetary value, the two room options presented at the beginning of each block differed in terms of whether the participant or a computer algorithm selected between the two actions available in the chosen room on trials within the block. At the beginning of the experiment, participants were instructed that, in each block, they would have the option of playing in a room themselves or having the computer play for them. They were further told that, if they choose to have the computer play, the computer would select each available action in the room equally often, by alternating between available actions across trials (e.g., A1, A2, A1, A2…). On the choice screen at the onset of each block (top of Fig. 2), the word “auto-play” was always printed below one room and the word “self-play” below the other room, to indicate whether the participant or the computer would be playing in that room for the duration of the block.

Since we were primarily interested in assessing whether there would be a preference for self-play when choosing between two high, but not between two zero, divergence rooms, a second difference from Experiment 1 was that a larger proportion of room choices involved two rooms that had the same divergence (either high or zero), as well as the same expected value, differing only in terms of self-play vs. auto-play. If self-play is valued only in high-divergence environments, then participants should select self-play over auto-play when choosing between two high-divergence rooms, but not when choosing between two zero-divergence rooms. In addition, we included several blocks in which participants choose between a high- and zero-divergence room, with either the high-divergence room or the zero-divergence room being the self-play option. We predicted that the preference for high-divergence, demonstrated in Experiment 1, would only emerge when the high-divergence option was also the self-play option. The remaining blocks were ones in which both expected value and divergence differed across self- and auto-play options.

## Results

### Pre-training on action-token probabilities

Participants required on average 2.08 (*SD* = 0.93) sessions of practice on the action-token probabilities. Mean probability ratings, obtained before and after the gambling phase, are shown in the bottom two rows of Table 2. As in Experiment 1, mean rated probabilities were very close to programmed ones, both prior to gambling and immediately following the gambling phase.

### Does a preference for high divergence depend on self- vs. auto-play?

The mean proportions of high- over zero-divergence choices, when the high-divergence option was self-play versus when it was auto-play, with expected values held constant across options, are shown on the left in Fig. 3b. Planned comparisons revealed that, as predicted, participants preferred the high-divergence over the zero-divergence room significantly more often when the high-divergence room was associated with self-play (and the zero-divergence room with auto-play) than when the high-divergence room was associated with auto-play (and the zero-divergence room with self-play), *t*(*23*) = *2*.*41*, *p* = *0*.*025*, *d*_*z*_ = *0*.*49*. Indeed, when the high-divergence room was auto-play and the zero-divergence room was self-play, selection of the high-divergence room did not deviate significantly from chance, *t*(*23*) = *0*.*11*, *p* = *0*.*914*, *d* = *0*.*02*. As in Experiment 1, we confirmed that these differences across options with identical expected values were not due to unintended differences in monetary earnings: Mean monetary earnings were the same for high-divergence self-play ($10.98 ± 0.93), high-divergence auto-play ($10.83 ± 1.49), zero-divergence self-play ($11.08 ± 0.94) and zero-divergence auto-play ($10.87 ± 1.35) rooms; t < 1.07 and p > 0.30 for all pairwise comparisons. For those few blocks in which both divergence and expected value did differ across self- and auto-play options, in either the same or opposite directions, there was a clear effect of expected value, such that the proportion of high-divergence choices was significantly greater when the high-divergence room was associated with greater expected value, whether it was an auto-play room (mean difference = 0.35; *t*(*23*) = *3*.*78*, *p* < *0*.*001*, *d*_*z*_ = *0*.*77*) or a self-play room (mean difference = 0.32, *t*(*23*) = *3*.*11*, *p* = *0*.*005*, *d*_*z*_ = *0*.*65*).

### Does a preference for self-play depend on divergence?

The mean proportions of self-play over auto-play choices, for blocks in which the divergence of both options was either high or zero, with expected value held constant across self- and auto-play options, are shown on the right side of Fig. 3b. Planned comparisons revealed that participants preferred self-play over auto-play significantly more often when choosing between two high-divergence rooms than when choosing between two zero-divergence rooms, *t*(*23*) = *2*.*18*, *p* < *0*.*039*, *d*_*z*_ = *0*.*45*.

## Discussion

We assessed the influence of instrumental divergence – the extent to which actions differ with respect to their outcome probability distributions – on behavioral preferences in a gambling task. In each round of gambling, participants chose between two pairs of actions, knowing that they would be restricted to the actions in the selected pair on subsequent trials in that round. One pair of actions had high instrumental divergence while the other pair had zero divergence. In Experiment 1, we found that, when other decision variables, such as expected value and outcome entropy, were held constant, participants chose the high-divergence option significantly more often than the zero-divergence option. Moreover, when expected values differed across options in either the same or opposite direction of divergence, choice performance deviated significantly from chance only when expected value differed in the same direction as instrumental divergence, suggesting that high-divergence choices were made at the expense of monetary gain. In Experiment 2, we used an “auto- vs. self-play” manipulation to rule out outcome diversity as a source of the behavioral preference for high instrumental divergence.

An important aspect of subjective utilities is that they tend to fluctuate from one moment to the next: Water is of great value when one is thirsty, but food is preferable when one is hungry; you may desire a cup of strong coffee in the morning but prefer a calming cup of tea in the evening; today you may be in the mood to indulge in a delicious piece of cake, but tomorrow you may have committed to a healthier lifestyle. Indeed, constantly changing consumer preferences is a topic of intense study in economic and marketing research. As noted, it is exactly because of such changes in subjective utility that flexible instrumental control is essential for reward maximization. Here, we have simulated dynamic fluctuations in utilities by sporadically changing the monetary values assigned to token outcomes throughout the task. It is possible that, had the monetary token values instead remained fixed, the clear preference for high instrumental divergence would have been reduced, or even absent. On the other hand, since, in the real world, subjective utilities are constantly changing, the preference for high instrumental divergence might be a stable psychological construct that governs decision-making across dynamic and static environments. Further research is needed to determine whether dynamic changes in outcome utilities are necessary for this preference to emerge.

Another important consideration is the greater perceptual diversity, or distinctiveness, of token outcomes in our high-divergence conditions. In a series of studies, Ayal and Zakay^10^ asked participants to choose among various “betting pools”, where the perceptual diversity of betting options varied (e.g., rolling the same dice three times vs. rolling a dice, then spinning a roulette wheel and then drawing a card), while the odds of winning on a given bet, and the monetary reward associated with a win, was held constant. They found a significant preference for the most perceptually diverse pool and demonstrated a trade-off between utility and diversification, such that the attempt to maximize outcome diversity led participants to prefer alternatives with lower expected utility (see^11^ for similar results). In our Experiment 1, the high-divergence option was also that with the greatest outcome diversity. In Experiment 2, we used an “auto- vs. self-play” manipulation to dissociate these variables. A particularly interesting aspect of our results is that, when flexible instrumental control over outcomes was removed, then so was the preference for greater outcome diversity: that is, when the high-divergence (and thus high outcome diversity) option was auto-play, we found no preference for that option. These results suggest that outcome diversity might derive its apparent value from its association with flexible instrumental control: when presented in close proximity to conditions with true flexible control, conditions with relatively high levels of outcome diversity, but without flexible control, lose their appeal.

Our self- vs. auto-play manipulation is also related to several recent studies that have contrasted conditions in which participants made voluntary decisions with conditions in which participants were forced to accept a computer-selected option, demonstrating a clear preference for stimuli associated with free- over forced-choice^12^,^13^,^14^. In those studies, the complete absence of choice when a computer makes the selection can be likened to a Pavlovian scenario, in which outcome states are signaled by perceptual cues, irrespective of any action taken by the agent. Here, in contrast, we explore the value of flexible control within the instrumental domain, defining free choice, not in terms of whether a decision is voluntarily made, but in terms of the extent to which such decisions have a meaningful impact on future states. This distinction between actual and meaningful choice has several important implications: First, when forced to accept a computer-generated decision, there is a potential discrepancy between the intended and forced selection that might generate aversive affect. Second, the complete absence of choice might trigger a reduction in attentiveness or concentration that, in turn, reduces subsequent processing of the valence of obtained outcomes. Finally, relative to no choice, free-choice might engage post-choice reevaluation processes aimed at reducing psychological tension stemming from consideration of the desirable features of the rejected alternative^15^,^16^. These potential sources of a preference for flexible control are all ruled out by the current design. Our results indicate that, in the absence of meaningful choice (i.e., in the zero-divergence condition), the preference for free-choice over a computer-generated selection (i.e., for self- over auto-play) is significantly reduced.

An important consequence of assessing levels of flexible instrumental control across voluntary decisions is that it allows us to consider implications for different action selection strategies. Theories of instrumental behavior distinguish between goal-directed actions, motivated by the current probability and utility of their consequences, and habitual actions, which are rigidly and automatically elicited by the stimulus environment based on their reinforcement history. Although computationally expensive^17^,^18^,^19^, the on-the-fly construction of goal-directed action values allows for flexible adjustment in the face of changing circumstances. However, when instrumental divergence is low, or zero, the greater processing cost of goal-directed computations does not yield the return of flexible control, suggesting that a less resource-intensive, habitual, action selection strategy might be optimal. Intriguingly, evidence from the rodent literature^20^,^21^ suggests that a goal-directed strategy dominates when alternative actions yield distinct sensory-specific outcomes (i.e., when instrumental divergence is high). Consistent with such demonstrations, Liljeholm *et al*.^22^ found greater sensitivity to sensory-specific outcome devaluation – a defining feature of goal-directed performance – when each action alternative yielded a distinct sensory-specific outcome than when the probability distribution over outcomes was the same across actions. Further assessment of the role of instrumental divergence in the arbitration between goal-directed and habitual decision strategies is an important avenue for future work.

Another phenomenon closely related to our findings is that of *learned helplessness* – a lack of exploration, and failure to exercise instrumental control, following exposure to uncontrollable events. In a classic initial demonstration, Seligman and Maier^23^ found that, following exposure to inescapable shock, dogs failed to escape shock that was in fact avoidable. Subsequent studies replicated these findings in the domain of human problem solving, showing that participants were less likely to successfully solve anagram problems following pre-treatment with unsolvable problems^24^. More recent research has investigated the role of reward prevalence in reduced exploration following exposure to uncontrollable events^25^. The current studies differ from previous work on learned helplessness in two critical respects: First, rather than differences in exploratory behavior, we are assessing a behavioral preference for environments with high instrumental divergence, thus evaluating the intrinsic value of flexible instrumental control. Second, even in our zero-divergence conditions, participants were able to obtain monetary outcomes by performing instrumental actions. In contrast, conventional induction of learned helplessness entails a complete absence of instrumental contingencies. Further work is needed to determine how degrees of exploratory behavior scale with differences in instrumental divergence.

Notably, learned helplessness, and a perceived lack of control over negative outcomes more generally, has been strongly linked to depression^26^,^27^, and has been shown to predict dysphoric and anxious symptoms^28^. Indeed, an aberrant experience of voluntary control, or “sense of agency”, appears to be a common characteristic of psychiatric illness: Schizophrenic individuals differ from healthy controls both in the degree of intentional binding – a perceived compression of the time interval between voluntary actions and their consequences – and in reported self- vs. external attributions of decision outcomes^29^,^30^,^31^,^32^,^33^. Although operational definitions of agency and volition differ substantially across these reports, and while related research suggests that schizophrenic and depressed individuals may be more fundamentally impaired with respect to goal-directed learning and performance^34^,^35^, the apparent role of instrumental choice in psychopathology suggests that a better understanding of the perceived value of flexible instrumental control in healthy individuals may be of significant clinical interest.

Finally, at the neural level, previous work has implicated the inferior parietal lobule (IPL) in several aspects of goal-directed processing, including the computation of instrumental contingencies^36^,^37^ the attribution of intent^38^ and the sense of agency^39^,^40^,^41^. Structurally, the rostral region of the supramarginal gyrus of the IPL, cytoarchitectonically distinct from more caudal areas^42^, has been shown to be heavily connected to inferior frontal and premotor cortices^43^,^44^,^45^,^46^; regions known to play a prominent role in voluntary action selection^47^, as well as in self-attribution^48^ and the sense of agency^49^. Consistent with this functional and structural anatomy, using a simple value-based decision-making task, Liljeholm *et al*.^50^ found that activity in the rostral right IPL scaled with instrumental divergence. However, critically, the task employed by Liljeholm *et al*. did not allow for an assessment of a behavioral preference for high instrumental divergence nor for investigation of a common neural code for divergence and reward. Notably, when directly assessing neural activity during anticipation of free choice, Leotti and Delgado^13^,^14^ found that activity in the ventral striatum, an area frequently implicated in reward anticipation and reward prediction-errors^51^,^52^, was greater for a cue that indicated an upcoming free-choice trial than for a cue signaling a no-choice trial. Further work is needed to determine how neural representations of the information theoretic, “cognitive” aspects of instrumental divergence may interact with those subserving affective and motivational processes; an integration implied by the behavioral preference for high instrumental divergence demonstrated here.

In conclusion, we have introduced a novel decision variable – instrumental divergence – and demonstrated its influence, dissociable from that of other motivational and information theoretic factors, on behavioral choice preference. Complementing previous work on the diversity^10^,^11^ and controllability^25^,^53^,^54^ of decision outcomes, our results contribute toward a fuller characterization of goal-directed cognition and action.

## Additional Information

**How to cite this article**: Mistry, P. and Liljeholm, M. Instrumental Divergence and the Value of Control. *Sci. Rep*. **6**, 36295; doi: 10.1038/srep36295 (2016).

**Publisher’s note:** Springer Nature remains neutral with regard to jurisdictional claims in published maps and institutional affiliations.

## Figures and Tables

**Table 1:** Token probabilities and reward distributions, gambling rooms and choice scenarios in Experiment 1.

	Token Outcomes			Rooms		Choice scenarios	
	*blue*	*green*	*red*	High Div.	Zero Div.	a vs. e	d vs. e
A1 & A2	0.0	0.7	0.3	a. A1 & A3	e. A1 & A2	a vs. f	d vs. f
A3 & A4	0.7	0.0	0.3	b. A2 & A4	f. A3 & A4	b vs. e	a vs. b
Balanced	$2	$2	$1	c. A1 & A4		b vs. f	e vs. f
Unbalanced 1	$1	$2	$3	d. A2 & A3		c vs. e	
Unbalanced 2	$2	$1	$3			c vs. f	

The top two rows in the 2nd column indicate the probability of each colored token given either of the actions listed to the left; the bottom three rows indicate the monetary value of each token in balanced and unbalanced blocks. The third column shows the pair of actions available in each room, and the fourth column the combination of rooms into choice scenarios.

**Table 2:** Mean ratings of token probabilities following pre-training, for programmed probabilities of 0.7, 0.0  and 0.3, obtained before and after gambling, in Experiments 1 and 2.

		0.7	0.0	0.3
Exp. 1	Before	0.70 ± 0.02	0.00 ± 0.02	0.30 ± 0.02
	After	0.64 ± 0.16	0.04 ± 0.15	0.31 ± 0.09
Exp. 2	Before	0.69 ± 0.02	0.00 ± 0.02	0.30 ± 0.03
	After	0.64 ± 0.16	0.05 ± 0.16	0.32 ± 0.09
